# Ten simple rules to bridge ecology and palaeoecology by publishing outside palaeoecological journals

**DOI:** 10.1371/journal.pcbi.1012487

**Published:** 2024-10-15

**Authors:** Nick Schafstall, Xavier Benito, Sandra O. Brugger, Althea L. Davies, Erle Ellis, Sergi Pla-Rabes, Alicja Bonk, M. Jane Bunting, Frank M. Chambers, Suzette G. A. Flantua, Tamara L. Fletcher, Caroline Greiser, Armand Hernández, Benjamin Gwinneth, Gerbrand Koren, Katarzyna Marcisz, Encarni Montoya, Adolfo Quesada-Román, Amila S. Ratnayake, Pierre Sabatier, John P. Smol, Nancy Y. Suárez-Mozo

**Affiliations:** 1 Czech University of Life Sciences, Prague, Czech Republic; 2 Marine and Continental Waters Programme, Institute for Agrifood Technology and Research (IRTA), La Ràpita, Spain; 3 Department Umweltwissenschaften, University of Basel, Basel, Switzerland; 4 Paul Scherrer Institute, Villigen, Switzerland; 5 School of Geography and Sustainable Development, University of St Andrews, St Andrews, United Kingdom; 6 Department of Geography and Environmental Sciences, University of Maryland, Baltimore County, Maryland, United States of America; 7 Universitat Autònoma de Barcelona, Bellaterra (Cerdanyola del Vallès), Catalonia, Spain; 8 School of Geography, Environmental Management and Energy Studies, University of Johannesburg, Johannesburg, South Africa; 9 Department of Geomorphology and Quaternary Geology, University of Gdańsk, Gdańsk, Poland; 10 School of Environmental Sciences, University of Hull, Hull, United Kingdom; 11 Centre for Environmental Change and Quaternary Research, University of Gloucestershire, Cheltenham, United Kingdom; 12 Department of Biology, University of Bergen and Bjerknes Centre for Climate Research, Bergen, Norway; 13 School of Physics, Chemistry and Earth Sciences, University of Adelaide, Adelaide, Australia; 14 School of Earth and Environment, University of Leeds, Leeds, United Kingdom; 15 Department of Physical Geography and Bolin Centre for Climate Research, Stockholm University, Stockholm, Sweden; 16 Department of Forest Ecology and Management, Swedish University of Agricultural Sciences, Umeå, Sweden; 17 Departamento de Física e Ciencias da Terra, Centro Interdisciplinar de Química e Bioloxía, Universidade da Coruña,Coruña, Spain; 18 Département de géographie, Université de Montréal, Montréal, Canada; 19 Copernicus Institute of Sustainable Development, Utrecht University, Utrecht, the Netherlands; 20 Climate Change Ecology Research Unit, Adam Mickiewicz University, Poznań, Poland; 21 Geosciences Barcelona, CSIC, Barcelona, Spain; 22 Escuela de Geografía, Universidad de Costa Rica, San José, Costa Rica; 23 Department of Applied Earth Sciences, Uva Wellassa University, Badulla, Sri Lanka; 24 EDYTEM, Université Savoie Mont Blanc, CNRS, Le Bourget du Lac, France; 25 Paleoecological Environmental Assessment and Research Lab (PEARL), Department of Biology, Queen’s University, Kingston, Canada; 26 Intituto de Ciencias del Mar y Limnología, Universidad Nacional Autónoma de México, Ciudad de México, Mexico; Dassault Systemes BIOVIA, UNITED STATES OF AMERICA

## Abstract

Owing to its specialised methodology, palaeoecology is often regarded as a separate field from ecology, even though it is essential for understanding long-term ecological processes that have shaped the ecosystems that ecologists study and manage. Despite advances in ecological modelling, sample dating, and proxy-based reconstructions facilitating direct comparison of palaeoecological data with neo-ecological data, most of the scientific knowledge derived from palaeoecological studies remains siloed. We surveyed a group of palaeo-researchers with experience in crossing the divide between palaeoecology and neo-ecology, to develop Ten Simple Rules for publishing your palaeoecological research in non-palaeo journals. Our 10 rules are divided into the preparation phase, writing phase, and finalising phase when the article is submitted to the target journal. These rules provide a suite of strategies, including improved networking early in the process, building effective collaborations, transmitting results more efficiently to improve cross-disciplinary accessibility, and integrating concepts and methodologies that appeal to ecologists and a wider readership. Adhering to these Ten Simple Rules can ensure palaeoecologists’ findings are more accessible and impactful among ecologists and the wider scientific community. Although this article primarily shows examples of how palaeoecological studies were published in journals for a broader audience, the rules apply to anyone who aims to publish outside specialised journals.

## Introduction

Like any other science, the field of ecology encompasses numerous disciplines, each fostering and sustaining a diverse array of specialist journals. Trends toward methodological specialisation within disciplines are far from uncommon [1], and as a discipline within ecology, palaeoecology is no exception to this. Drawing on insights from many fields and disciplines, including biology, chemistry, geography, geology, climatology, and archaeology, palaeoecology offers challenging, yet exciting cross-disciplinary approaches focused on understanding long-term ecological patterns, processes, and dynamics under natural and human forcing [2–3]. Palaeoecology addresses research questions and frequently engages with concepts common to both applied and fundamental ecological research such as restoration, human legacies, bioindicators, climate change, and community dynamics [4]. However, palaeoecology is methodologically distinct, using proxy-based records to reconstruct past environments on longer timescales than is possible through direct observations, and, hence, it has cultivated a suite of techniques and a terminology that are unfamiliar to many neo-ecologists (ecologists working with data based on direct observations, with records generally not older than a few decades) (see Table 1).

**Table 1:** Explanations of the terminology and jargon used in the text.

Term	Explanation
Age-depth modelling	For a series of dated samples, the relationship between age and depth is statistically modelled. Bayesian models that interpolate probabilistic age on a linear projection between the dated samples, based on an average sediment accumulation rate, are the most commonly used approach.
Anthropocene	A term that recognizes significant human impacts on Earth’s geology, landscape, limnology, and ecosystems, including, but not limited to, anthropogenic climate change.
Neo-ecology	“Modern” ecology, based on direct observations ranging from days to the last few decades.
Palaeoecology	The study of the relationships between ancient organisms (e.g., plants, animals, bacteria, fungi) and their environments. Palaeoecology can provide information about ecology on timescales of many thousands of years, and in time resolutions ranging from sub-annual to centennial.
Palaeo-archives/palaeo-records	Geological (e.g., sediment cores, speleothems) and biological (e.g., tree rings, corals) materials that preserve evidence of past environmental changes.
Palaeoclimatology	The scientific study of climates predating the extensive use of meteorological instruments, by using proxies from palaeo-records to reconstruct climatic variables.
Palaeo-community	The community of researchers who investigate environmental records using palaeo-methods.
Palaeogeography	The study of historical geography, generally the history of physical landscapes.
Palaeolimnology	The discipline that studies long-term changes in lakes, based on palaeo-records.
Palaeo-methods	A suite of methods enabling the recovery of ecological data from sedimentary archives and other natural sources, including the retrieval of such archives, dating of archives, extraction, the identification and quantification of biotic remains and proxies, and interpretation of such data to reconstruct aspects of past ecologies and ecosystems.
Palaeo-perspective	The long-term perspective derived from palaeoecological records on (changes in) environmental properties such as climate, vegetation, nutrients in a lake, and more. Information to inform the palaeo-perspective is collected from palaeo-records.
Proxy	A proxy (or proxy variable) yields clues as to temperature, precipitation, productivity, or other environmental conditions. Examples of proxies are the presence and absence of fossils; the abundance ratios of fossils; and the chemical composition of fossils, geophysical properties of sediments, growth rings of trees and corals.
Quaternary timescale	A period of centuries to millennia within the last 2.6 million years that includes ecological phenomena of species that are extant today.
Sample dating	An important palaeo-method, based on the radioactive decay of lead (in the case of material covering maximum last 120 years), carbon (in the case of material younger than ca. 50,000 years), or other isotopes (^238^U, ^235^U, and ^232^Th) for longer timescales.
Sediment	Particulate material transported or in situ precipitated and deposited in another location (in the same lake or basin, or further away). Sediment can consist of rocks, sand, or clay particles, as well as the organic and inorganic remains of living organisms.
Site	Location where palaeo-records were retrieved, such as a lake, sediment outcrop, peatland, tree, or coral reef.
Stratigraphy	Layer arrangement of accumulated soil, sediment, or rock. Stratigraphy is typically used to estimate the relative age and origin of a sediment layer or to correlate sediment layers of the same or different sites to each other.
Taphonomy	The study of what happens to organic remains (plants, animals, or others) once the organism dies, how it ends up in the sediment, and what processes alter the remains after deposition.

**Fig 1:**
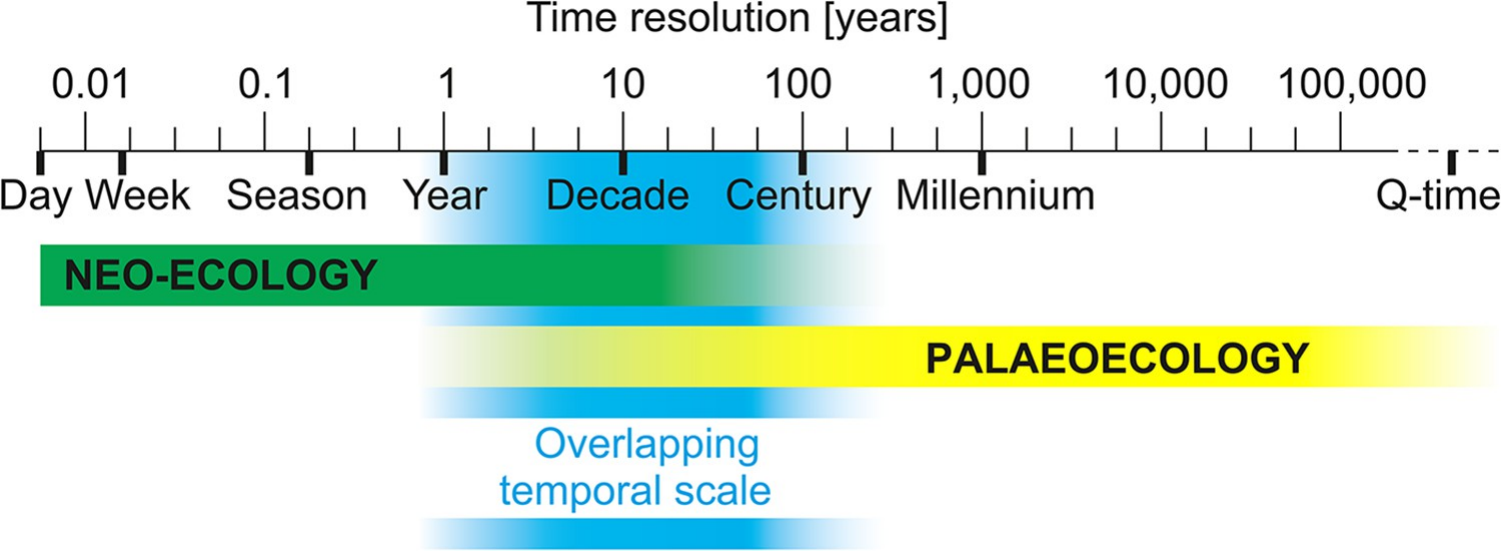
Palaeoecology can show the variability of species assemblages and ecosystems over a longer timescale (e.g., 10,000 years or even further in Quaternary time), but certain proxy records can provide decadal or yearly resolution and overlap with the temporal resolution used in neo-ecological studies. Adapted from [3].

These “palaeo” methodological obstacles, summarised in[5–6], include (1) technical barriers (such as lack of time to engage in interdisciplinary collaborations and challenges/difficulties in translating unfamiliar information); (2) a lack of awareness and/or limited access to the methodology and associated publications; and (3) preconception barriers that hinder the willingness of other diverse audiences to consider unfamiliar types of evidence, since palaeoarchives may produce evidence in formats not directly applicable to neo-ecology, management, and policy. This preconception barrier between diverse audiences and palaeo-methods and the time constraints on engaging with these audiences often lead to the classification of palaeoecology as a separate field rather than a discipline within ecology [3]. Progress in effective collaboration through open science [7] and computational palaeoecology (e.g., [8–10]), as well as adopting approaches that have so far been applied mainly for ecological studies (e.g., the use of organismal functional traits; [11–12]), have enhanced the capability to integrate and compare long palaeo-records of microbiota, plants, animals, and abiotic factors, on a Quaternary timescale [13] with directly observed modern records (data spanning the last 50 years or less [14]) (Fig 1). Nevertheless, more effort and input are needed from palaeo-researchers to integrate palaeoecology within the broader field of ecology sufficiently to include a “palaeo-perspective” in scientific discussions about present and future environmental challenges [15].

A key challenge for palaeoecology is securing publication in outlets intended for neo-ecological audiences, as this is an important means of building and maintaining cross-disciplinary connections with neo-ecologists. Conversely, the concept of “belonging”, which has been used to explain the attraction of publishing in specialised journals [16], reinforces the boundaries between palaeo- and neo-ecology. Therefore, the challenges related to synthesising and cross-fertilizing knowledge persist, even though overcoming them often leads to multiauthored papers that contribute meaningfully to the understanding of “wicked” problems (here, complex environmental problems with seemingly irreconcilable interpretations for stakeholders; see the discussion in [17]). Such cross-disciplinary publications can be very effective in transferring knowledge between different disciplines within ecology [18–19]. Currently, a growing number of palaeoecologists, especially early-career researchers (ECRs), are searching for practical guidance to aid the dissemination of palaeoecological knowledge for uptake in the broader field of ecology [14]. In the current era of access to increasing open long-term ecological data, analytical methods, and global collaborations, ECRs are well positioned to elevate the applicability and incorporation of data from palaeo-research. However, palaeoecology is dominated by researchers from the Global North [20], and non-English ECR palaeoecologists might face additional challenges due to language barriers [21]. Here, we offer “Ten Simple Rules” as guidelines to facilitate publishing in journals outside the discipline of palaeoecology, supported by detailed examples and exemplar references. Many of the referenced publications can be found in the British Ecological Society Palaeoecology Special Interest Group list of influential papers in palaeoecology [22].

### Methodology and target audience

**Fig 2:**
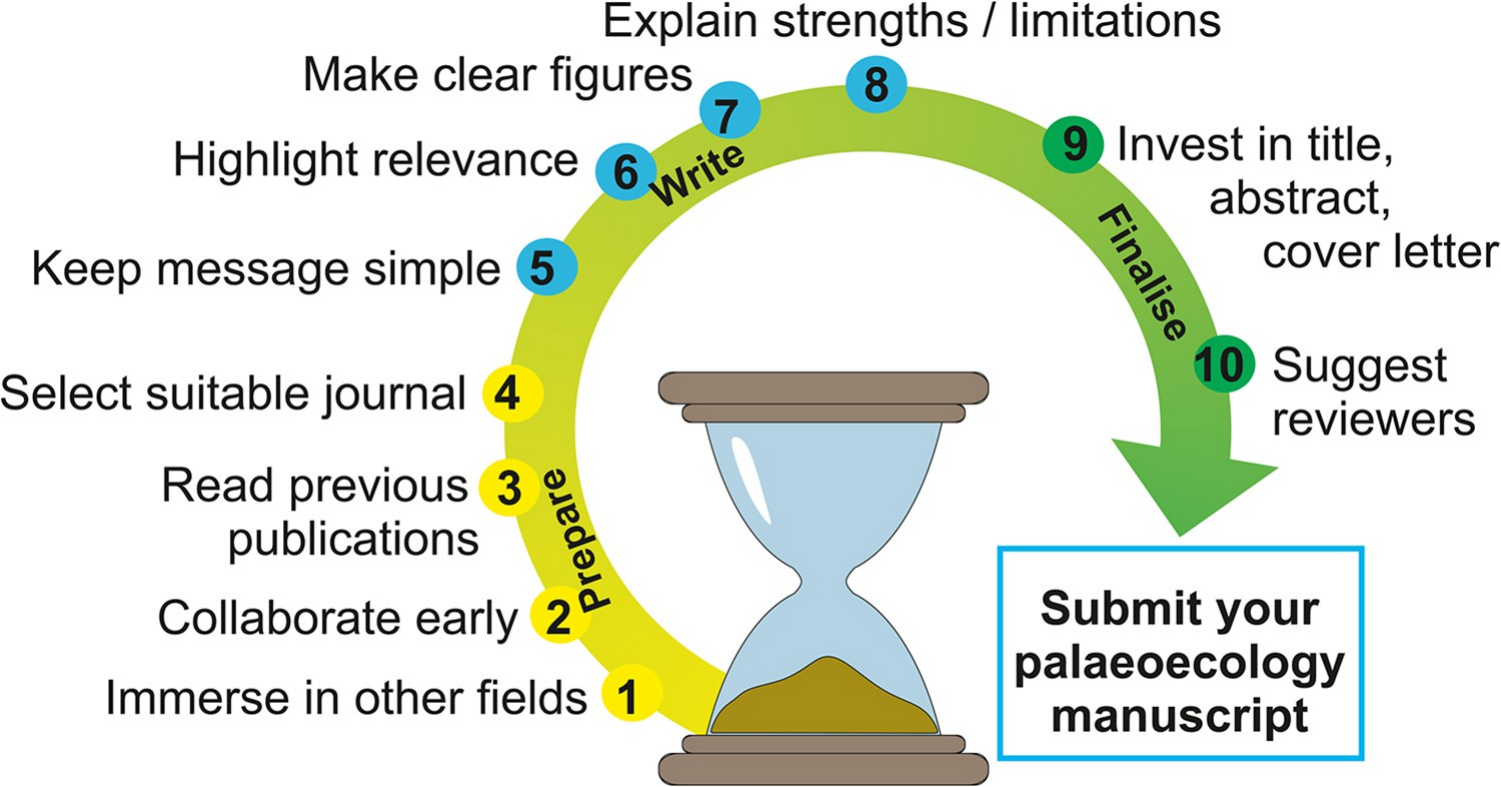
Ten Simple Rules to publish data from a specialised subfield (palaeoecology) in a journal aimed at a broader readership (interdisciplinary ecology). The 3 colours within the arrow represent the 3 main phases of preparation, writing, and finalising your article.

The guidance presented here was collaboratively developed with input from the wider palaeo-community. Initially, a call was sent to various communities of palaeo-researchers soliciting guidelines that they would recommend to ECRs aiming to publish their research outside specialist palaeo-journals. The call was initiated by the 2 lead authors of this paper and was sent to mailing lists with wide geographic membership and spanning a range of proxies within the palaeoenvironmental sciences remit (e.g., aquatic, terrestrial, ecological, geological, and climatic). We received responses from 45 contributors from various parts of the world and with differing backgrounds, which included suggestions, guidelines, and recommended readings. The instigating ECRs thematically grouped these contributions into topics and added explanatory subtext to these topics based on the input of the contributors. Subsequently, all contributors were invited to review and comment on the recommendations to refine them into “simple rules” on how to publish outside one’s own research specialisation. The original contributions (S1 File) were structured into 10 broad thematic guidelines (“Ten Simple Rules”), arranged chronologically from designing a study, to writing a manuscript, and ultimately submitting the manuscript to a scientific journal outside the field of palaeoecology (summarised in Fig 2). The examples provided here focus on publishing palaeoecological work to reach a wider ecological audience.

### Rule 1: Immerse yourself in research fields other than palaeoecology

Engage in active learning about fields or disciplines you are not trained in by reading publications and upper-level textbooks or participating in relevant courses, seminars, or conference sessions. Such activities can help you identify “hot topics,” emerging technologies, and pressing questions in your target field. Several publications offer an overview of how palaeoecology can be applied to address questions in ecology, touching upon topics such as lake restoration (e.g., [23]), moorland management (e.g., [24]), or browsing pressure by herbivores (e.g., [25]), and more general issues in science policy (e.g., [26]). To identify how palaeoecology can best contribute to a broader understanding of biosphere functioning, [2] codeveloped a list of 50 priority research questions.

### Rule 2: Start collaborating as early as possible with colleagues outside of the palaeo-community

Building effective collaborations with researchers from other fields and disciplines is critical for connecting your specialisation to other disciplines and extending the reach of research beyond individual fields. Consider joining a working group in an area of shared interest to find collaborators and contribute to existing efforts as early as possible in your career. Engaging with scientific societies that foster cross-disciplinary networking and interdisciplinarity in palaeosciences (e.g., British Ecological Society Special Interest Groups, Past Global Changes (PAGES) working groups) can help initiate collaboration and lead to codesigned research questions that generate improved understanding across palaeoecology, neo-ecology, and other disciplines. Some connections may not result in a sustained working partnership, in projects or publications, but can still be a valuable part of the learning process. Examples of fruitful multidisciplinary collaborations include the combination of archaeological records with palaeoecological records to measure the past impact of humans on their surrounding landscapes [27–28], cross-community efforts to model the responses of past civilisations to past ecological, climatic, and environmental changes [29], and analysing modes of climate variability through the Holocene [30]. Such studies have generated deeper insight into the functioning and interactions of climatic, ecological, and social systems.

### Rule 3: Learn from previous palaeoecological publications in ecology journals

Examine the word choice, writing conventions, and style of articles in the journals where you would like to publish. It is valuable to examine which palaeoecological articles published in ecology journals have been cited by ecologists to identify examples that have achieved traction in the wider field, rather than those that are popular within palaeoecology. Collaboratively published articles in an applied ecology journal may be particularly successful, gaining many citations from both palaeoecologists and neo-ecologists over many years following publication (e.g., [31]). Regardless of the journal, you can dissect how the data and main messages are communicated as a model for improving your field-targeted writing. For example, [32] approached how palaeolimnology could be used in biodiversity studies by defining widely used ecological concepts in their palaeoecological context (e.g., temporal beta diversity, functional diversity), while [33] review stratigraphic expressions that could mark the Anthropocene transition using sentinel remote lakes. [34] investigated concepts from complex dynamic theory such as regime shifts, by applying palaeo-community time-series approaches. Additionally, you can broaden your knowledge by exploring disciplines that share (aspects of) methodology and terminology with palaeoecology, such as palaeoclimatology and palaeogeography. Publications from the disciplines of palaeoclimatology (e.g., [35,36]) and palaeogeography (e.g., [37]) are frequently successful in reaching a wider readership beyond their respective fields and could be used as examples for reaching a wider audience with your palaeoecological publication.

### Rule 4: Target appropriate journals that have previously published palaeoecological content

Inter- and multidisciplinary journals (e.g., *PNAS*) might be more receptive to your manuscript than highly specialised ecological journals. Alternatively, a specialised journal outside your research field, but dedicated to your geographical region (e.g., *Arctic* and *Austral Ecology*), study system (e.g., *Journal of Limnology*), or environmental processes (e.g., *Nature Climate Change*) may be open to your work. Journal choice will affect the approach taken in the manuscript to convey the main idea to the journal’s audience, so should be made at an early stage in writing. If this is your first article for a wider audience, we recommend targeting a journal that has previously published articles in your specific field, increasing the likelihood that your manuscript will be received with an open mind and gain a more positive reception. The choice of journal is important since it determines how the study is presented (e.g., choice of language, formatting of diagrams) and what level of detail is appropriate. For example, writing a palaeoecology article with a focus on biogeography [38] will require a different emphasis on details about methodology and results than one for pollen specialists (e.g., [39]). Likewise, palaeolimnological findings can be published for limnologists (e.g., [40]), palaeoclimatologists (e.g., [41]), or a broader Earth system readership [42–43], and each different readership could be interested in different details from your study. Many journals accept presubmission inquiries to evaluate the manuscript’s fit, so you could contact the chief editor to clarify any uncertainties. It could also be rewarding to send a draft of your manuscript to someone in the readership of your target journal, asking if they would find your manuscript appropriate for that journal and whether your draft contains the information that they would expect (Rules 5 to 7).

### Rule 5: Keep the message simple for a non-palaeoecology audience

Once you start writing, keep the message clear and direct. Defining how your work relates to topics of relevance to the journal audience is crucial at an early stage in writing. Avoid lengthy descriptions of methods, results, or issues (e.g., taphonomy, age-depth modelling) that are not specifically needed to communicate the main message you wish to convey. Such specialised details can find a good home in the supporting information to maintain appropriate messaging in the main text for a broader audience. Keep terminology consistent throughout the manuscript, and ensure that key concepts are clearly defined, or provide a glossary with the terminology used in your manuscript [44]. This is particularly important if terms used differ from accepted (neo-)ecological terminology and definitions owing to the nuances of palaeo-data (e.g., spatial scales, timescales). Multiple studies exist in the literature as model examples from which to learn. For example, [45] offer a concise analytical account of the methods and findings of the complex numerical analyses implemented to apply their space-for-time substitution approach in the main text, while reserving some finer details for the supporting information. [46] quantified human-induced species extinction in a straightforward manner and provided an excellent example of a study that applies conceptual models to shed light on ecological theory using palaeo-data, of wide interest, for example, to conservation ecologists. Strengthening the applicability of this rule to palaeoecology will benefit from conveying “time” as a continuum regardless of subdiscipline methodological constraints as seen by researchers and practitioners outside the palaeo-community [47].

### Rule 6: Highlight the importance, relevance, and application of your palaeoecological research

After identifying key research questions and knowledge gaps in your target field (Rule 1), state clearly how your palaeo-perspective adds value to this theme. A common starting point is to set out how the longer timeframes provided by palaeosciences contribute to knowledge developed from (modern) shorter environmental or ecological time series (Fig 1). When palaeoecology focuses on millennial timeframes, integration with shorter ecological datasets that are often characterised by higher temporal resolutions can pose challenges (Fig 1), especially if the manuscript intends to offer management or policy recommendations. Reference to high-resolution examples using multidecadal sampling intervals or even annually laminated sediment records can be used to bridge the temporal gap between neo- and palaeoecological studies [48–50] and support the choice of methods and longer timescales. An integral aspect of highlighting the relevance of your methods could be the choice of your site and proxies. For example, a pollen record from a small lake records the general changes in vegetation in a radius of several kilometres around the site [51]. The plant seeds from that same site record local changes in plant species (growing on the site or within a few metres of the margins), and other types of fossil or biogeochemical proxies can contribute to reconstructing the environmental conditions under which those plant species persisted [52]. Integration of various palaeoecological proxies can deliver a comprehensive view of past environmental changes that, when combined with an understanding of management issues, can be used to develop management guidelines and environmental protection measures for certain ecosystems, for example, in terms of climate change scenarios [53–55]. Alternatively, studies can focus on the contrast—what long timescales provide that high-resolution, modern studies cannot [56]. Various concepts have been employed to underscore the relevance of palaeoecology in general ecology journals, such as identifying reference baselines to assess the degree of impact on ecosystems, filling knowledge gaps about introduced species, establishing long-term system trends to inform biomonitoring programmes or disentangling natural fluctuations in mean climate states from human-altered environmental regimes. These themes are well established, with numerous examples, including studies of the feasibility of monitoring aquatic diversity and human impact on the diversity using palaeoarchives [57], patterns of tree succession from pollen records to inform forest ecology [28], studies of glacial microrefugia that play a key role in conserving biodiversity [58], and biomonitoring to assess how measuring the resilience of ecosystems could be improved [59].

### Rule 7: Provide clear explanatory figures for non-palaeoecologists

Effective and clear visualisation of your research approach, methods, results, and implications will help editors, reviewers, and especially readers outside your research field to understand the value of your palaeo-contribution. There is a wealth of general literature on data visualisation, and a recognised shortage of scientific training in this area [60]. Prioritise visualising concepts and results that may be unfamiliar to some audiences, such as stratigraphic plots and age models, regardless of whether they are in your manuscript or the supporting data. Providing explanatory workflows and interpretations, especially when different numerical or statistical methods are applied, can also improve the readability of your paper (e.g., [44,61]). Multivariate palaeoecological diagrams are a complex form of data display that can benefit from creative rethinking and innovative approaches to visualisation, or from the simplification possible by effective use of tables to pull out the key features of interpreted palaeoenvironments. For example, [62] provided conventional multivariate stratigraphic diagrams alongside infographics to highlight key ecological shifts through time. Using both explanatory and exemplary figures, [63] generated community response diagrams that show nonequilibrium dynamics between plant functional responses and Holocene warming. [38] illustrate global island pollen trends following human arrival, aiding a broader audience in understanding the relevance of complex pollen datasets in support of the overarching biodiversity focus of the paper.

### Rule 8: Be clear about the strengths and limitations of your palaeo-data

Clearly and transparently acknowledging the strengths and limitations of your palaeo-data will enhance the value of your manuscript in the eyes of the editor, reviewers, and the journal’s audience. This acknowledgment should highlight the unique benefits offered by palaeo-data (e.g., using its long-term perspective for establishing baselines for species or whole ecosystems to compare with neo-ecological data; see Rule 6). However, making the limitations of paleo-data explicit (e.g., temporal resolution, spatial and environmental bias of sites) also gives potential collaborators or data users realistic expectations and demonstrates that you recognise the limits of your data. Recent data syntheses have been published in parallel with protocols to assess biases and uncertainties when analysing palaeoecological data [14,64]. In other cases, clear documentation of the data adjustments needed to combine spatially distant records and allow appropriate statistical analysis of the joint dataset has been reviewed positively (e.g., [37]). Moreover, acknowledging the conceptual barriers to the integration of palaeo-data with environmental sciences maintains rigour in the field [65]. Relevant examples include [66,67], which assess how terrestrial palaeoclimate records inform contested theories on past climatic variability and biotic evolution in South America. Similarly, [68] examine whether ecological processes have fundamentally changed during the Anthropocene. To do so, they offer examples grounded in theory to identify and bridge temporal mismatches between ecological and palaeoecological datasets. As palaeoecological analytical methods advance (e.g., [69,70]), more use can be made of existing data. Meanwhile, efforts to deposit palaeo-records in open databases facilitate their reuse and synthesis. For example, the online Neotoma Palaeoecology Database currently includes palaeo-records from more than 20,000 sites worldwide [71]. It covers time periods from centuries to millennia, often on a decadal time resolution, and includes a range of proxies from biotic remains and geochemical data.

### Rule 9: Make the title, abstract, and cover letter clear and compelling for a non-palaeoecology audience

The cover letter will likely be the first part of your manuscript that is scrutinised by the editor of the journal. State clearly how your palaeo-approach fulfils and aligns with the interests of the target journal’s readership and explain how your study’s findings address a critical research gap within the aims and scope of the journal, justifying the wider ecological or neo-ecological community for which your study is intended. Present informative counterarguments to previously published results [72], highlight any advances in the state-of-the-art [73,74], introduce novel ideas that may be unfamiliar to this readership [75], or stress interdisciplinary implications of palaeoecology (e.g., to archaeology and historians; [76,77]). Remember that for subscription-based journals (non-Open Access), the title, abstract, and keywords are the only components freely available and are the resources used by search engines. Test your draft title and abstract with colleagues from different fields and consult available guidance on getting published, both from the target journal’s guidelines and from freely available guides (e.g., [78]). Examples of cover letters are not readily available, so ask your peers, supervisors, or collaborators to share examples of a “winning” cover letter. The writing of an abstract is central to the manuscript and should not differ from an abstract for a palaeo-journal, providing a succinct summary of your article (i.e., aims, context, key findings, and conclusion; [79]) and incentivising the reader to continue reading. However, an eye-catching title could increase the readership of your work as more potential readers will open your article to see what it is about. Some examples of articles that invite further exploration through a compelling title are “Ecological Restoration in the Light of Ecological History” [80], “Landscapes in time and space” [81], “Diversity in time and space: wanted dead and alive” [82], and “Ancient human disturbances may be skewing our understanding of Amazonian forests” [83].

### Rule 10: Suggest reviewers who are familiar with palaeo-research

After you have constructed the study with research questions relevant to neo-ecology, made an effort to keep the message simple, provided clear figures, included the relevance and possible limitations of your research, and written a compelling cover letter, there is one final thing you can do: strategically recommend reviewers during submission. Suggest individuals whose work is primarily non-palaeo-focused, but who are familiar with long-term or palaeostudies. These individuals should already understand the aims, advances, and limitations of your study, but also appreciate its value for neo-ecologists. These reviewers can see the value in your work and guide you in communicating it most effectively to a neo-ecological audience. You could also suggest experts in your field or study region who have previously published outside palaeo-journals. Consider the reviewing process a virtual conversation between your research and the wider community you want to engage with, with the reviewers as your collaborators. Their experience and critical insight can improve the clarity and accessibility of your manuscript, ultimately broadening its impact on the target audience [84].

## Conclusions

Even the most seasoned and highly cited cross-disciplinary scholars began as ECRs who had to master the art of publishing outside their own research discipline or field. Based on the personal experience of many of these (former) ECRs, the main take-home message from this set of Ten Simple Rules is the importance of remaining open and receptive to ideas and learning from a wide range of disciplines. This openness fosters the development of well-rounded scientists and aids in decision-making when publishing outside specialist, discipline-oriented (palaeo) journals is a high priority. When aiming for a publication in a neo-ecological or broader journal, the writing process may demand additional time and effort, particularly to fine-tune the messages and presentation to suit an audience that needs to be convinced of the merits of palaeo-data and palaeo-analysis. However, connecting ideas and evidence across different fields and disciplines can improve the quality of research and has the potential to advance the wider research field as a whole. Many theories and issues central to palaeoecology also find resonance in the wider field of ecology. Publishing palaeoecological studies in neo-ecological and interdisciplinary journals is a crucial step in generating and maintaining conversations across the methodological differences that divide palaeoecology and neo-ecology. Following these Ten Simple Rules can stimulate open-minded, cross-disciplinary conversations for palaeoecologists to ensure that their work is disseminated and understood by mainstream ecological scientists, as well as encouraging ecologists to challenge their own assumptions about the suitability and relevance of long-term palaeoecological records to ecological questions and applications. We expect that these simple rules will be useful for researchers in any field of science who aim to publish in journals that serve a broader audience.

## Supporting information

S1 File Original contributions by email (anonymised and with generalised layout). (DOCX)
