# Supplementary material for: Ten simple rules to bridge ecology and palaeoecology by publishing outside palaeoecological journals
Source: PLoS Comput Biol. 2024 Oct 15;20(10):e1012487. doi: 10.1371/journal.pcbi.1012487 (PMC11573137; doi:10.1371/journal.pcbi.1012487)
Supplement: S1 File — (DOCX) [file pcbi.1012487.s001.docx]

**S1_Supplementary data to manuscript ‘Ten simple rules to bridge ecology and palaeoecology by publishing outside palaeoecological journals’ by Schafstall et al.**

**Original statements from all contributions (C1:C39, anonymised and formatted)**

**C1**

- Keep in mind that readers and potential reviewers are not experts in "paleo" sciences most likely. Try to use consistent, clear language without sophisticated, field-limited vocabulary if possible.
- Do not assume that all terms are clear to everyone. If possible, describe complicated processes, cite relevant literature, explain formulas, etc. This also applies to using terms that may have different meaning depending on the field (I can't remember the example now, gosh... I know something fire-related has one meaning when talking about fire reconstruction and the other when you talk about forestry, but I can't recall what it was).
- Find a colleague outside of your field who would read your manuscript and give you feedback. Correct everything, which may need further explanation, being simplified, etc.

**C2**

- I have a tip - a collaboration with a researcher who is not a typical paleo-person. That worked quite well in my case.

**C3**

I am currently going through the review process on a paleo paper in a journal that typically has more neontological studies than paleo stuff. It seems to me that we can improve our relevance to modern-day conservation biologists by emphasizing the data we can produce that they cannot.

- Paleontological data represents the best way to quantify shifting baselines by including specimens from pre-human, pre-industrial and modern timescales.
- For some ecosystems, death assemblages can be more complete than life assemblages, for example with regard to cryptic taxa on reefs that are hard to collect in life.
- Paleoecological approaches such as stable isotope proxies can be used to provide historical data for under-studied areas that previously had lower research investment. By doing so, it empowers researchers outside of the global North and West to study ecological change in their regions.
- Fossil-modern comparisons are limited because they require interdisciplinary collaboration. Neontologists struggle to work with fossil data due to the knowledge base needed (preservation bias, time averaging, etc). The other direction is also true (paleo people generally are less connected to NGOs and other conservation organizations, are often unfamiliar with the data types more at the cutting edge in neontological literature).

**C4**

- My tip for getting the attention of non-palaeos is to frame the paper to focus on a question of modern interest, build up the answer in general terms, and use your palaeo as a 'case study' that demonstrates your answer; be question focused rather than observation focused. This might mean one paper in a modern ecology or climate journal using a case study based on multiple published papers in the paleo literature where all the palaeo-details can be laid out for that palaeo-audience and need not be rehashed in detail for non-palaeos.
- As an aside, rather than trying to influence other fields by publishing in their journals, I work directly with colleagues in modelling who have direct interaction with modern modellers or also apply the same models to the modern. My palaeo-environements are not direct analogues for the future, but they do inform modellers as to how their models are working in the 'deep time laboratory'. I don't think the best way to influence other fields is through their literature, but through direct collaboration.

**C5**

- I have published in a religious studies journal by writing a review of general societal change in response to climate - this was after a symposium of religion and climate. Some people in my department asked why I would publish in something like that and my reply is that there is a whole group of people reading my work now that previously would not have been.
- Publishing in different languages. My vulgarisation for a french language magazine (journal? it is indexed) was accepted and essentially the same as my first published chapter but written in french, and made more accessible. The name is Le Climatoscope and I can send a copy when it is out.
- Looking for broader context/implications such as framing your current research in a different way. I recently attended a workshop of the Resilience Alliance and now knowing different resilience principles plan to re-write the story of the Maya in those terms, making it accessible and interesting to a whole group of people.
- Re-packaging data into a separate publication, I use faecal stanol concentrations from modern lakes to ground truth a proxy for population change in the past. This is necessary and interesting for palaeo people but doesn't really have much use for people living in the areas I sampled. I plan to publish that data again as a technical report on lake pollution alongside PAH data.
- Apply for interdisciplinary scholarship or awards- if successful helps pull you out of the "track" of organic geochem, inorganic, limnology etc and associates you with broader multi-disciplinary

**C6**

I mainly work in paleoscience for 15 years from lake and lagoon sediments and I work from Holocene to the last century to understand the impact of climate and human forcing on landscape and different parts of the environment.

- From my point of view the best way to reach a larger audience outside our field is to work on the last century with really specific societal questions (pollution,ecology trajectories...), this framework also allows us to calibrate our sedimentary record with monitoring data. In the same way our study could have a broader audience if it was multidisciplinary. In that way our community provides the temporal framework to other colleagues (ecologist, chemist, social scientist...) to highlight their specific question.
- I also think that the concept of the Earth Critical Zone is very relevant to gather different scientific communities around environmental studies with a paleo perspectives approach opening a large new field of investigation.

**C7**

- One simple rule: Make paleo relevant to our current challenges and expected future ones by crossing the 'language barrier'. OR Make paleo relevant to risk analysis, climate services and climate finance.

For example, the California fires... risk analysis would say whether they are 1 in 2 year events or 1 in 50 year events. We can check this with paleo data. We can check if past landscape modification, like seasonal burning, kept fires at bay during similar high temperatures or longer periodicity changes like ENSO etc.

**C8**

- Pursue research questions that are relevant for modern environmental problems (or modern environmental management).

**C9**

My most recent paper presented a new 500 year marine paleo record to JGR: Oceans. This journal publishes very few paleo work, so I think its relevant to your project!

- Identify a debate in the modern system. A paleo perspective on a modern debate or unknown is an easy way in to a "non-paleo" journal. Your record may not solve the debate, but it is relevant. Highlight opportunities for future synthesis and integration among scientists/subjects.
- Its all in the letter to the editor. This is the opportunity to make bolder statements than you might make in the manuscript. Brag about the record's novelty. Getting past the "sent out for review" step is key to getting the feedback you need. You can also be explicit that you chose the journal to introduce your type of work to a new audience. Don't be afraid to name the audience(s)!
- Include broad and accessible intro and background. Too much jargon on your paleo specialty will shut the door to the new audience. Target your introduction and background to people unfamiliar with paleo. You might have to describe things that are already basic assumptions or "well-known" by you and your colleagues.
- If all else fails, identify the weaknesses in your record. If you think your work could have benefited from involvement of a physicist or biogeochemist, for example, target that journal and suggest specific reviewers. The review process may give you valuable ideas to make your work more relevant (even if it gets rejected!).

**C10**

Was really interested by your email about ECRs publishing outside of palaeo and one field that I think palaeoecology research can be applied to is restoration ecology. There are lots of summary palaeo papers that outline hypothetically how palaeoecology can aid restoration ecologists, but much fewer provide any actual information to people restoring and managing sites. My tip for people wanting to publish palaeo work in restoration ecology journals would be:

- Tip: Provide actionable advice.

It may seem obvious but in palaeo papers our outcomes rarely involve a definitive action to take place. If we can state for instance that a particular species has been present at a site for millennia but became locally extinct or underrepresented in the recent past, that is something that restoration practitioners can use. A great example is Mark Bush's 2014 paper 'Galápagos History, Restoration, and a Shifted Baseline' in the journal Restoration Ecology.

If proxy analysis can be undertaken in conjunction with pre-restoration research then our data can be used to help to establish restoration goals that contain a temporal aspect that ecology on its own lacks.

**C11**

- Link to observations for the last decades

I noticed that some of my colleagues tend to be less interested in ice cores, tree rings, or other paleo proxies. But they are more excited when these records are connected to the contemporary records that they work with (e.g. satellite observations). This is especially powerful when a trend can be detected in a ~20 year satellite record and can also be diagnosed from the paleo record.

- Highlight improved process understanding

Paleo science can help to better understand (and represent in models) slowly evolving processes. If you highlight that your (paleo)research can constrain these processes better, then the focus is less on the specific time period, but more on understanding process dynamics.

- Appeal to societal relevance

In some cases, events from the past can be made more interesting or appealing to a general audience by raising the question: "what if this would happen today?" An example is a spike of cosmic radiation that was detected in the years 774–775 (<https://www.nature.com/articles/nature11123>). I saw a presentation where the presenter started explaining in detail what would happen if our society would experience a similar event (massive impact on our society because of the collapse of telecommunication infrastructure). This worked really well to get the audience excited about his research (and he mentioned that this helped him get a prestigious grant).

**C12**

- Present species/community patterns in long-term data like long term natural experimental data – this way ecologists or biologists can relate their long-term observations to paleoecology.
- Use language/wording typical for the other discipline, e.g. terms like “ecological niche”, “species succession” in palaeoecological papers that you want to send to biological journal.
- Look at palaeo data from other perspective, for example add a functional aspect to your interpretations to interest ecologists or biologists.

**C13**

- Show an interest in learning about the other fields and communicate with scientists beyond your own specialised field - by attending conferences, by networking with people beyond your own field of expertise. This has worked relatively well for me as a paleontologist, but definitely easier because I come from another field originally.
- Read more broadly, beyond your own field of expertise and see how the methods used by others can be applied to your own discipline. Especially now that we are going towards big data analyses, there are several methods that can be transdisciplinary/interdisciplinary.
- I feel a lot of people tend to think that ONLY their field is important. This needs to change. Be open-minded.

**C14**

- #1 Non-paleo experiences: non-paleo are not interested in dating models. Put most of it in supplemental and only put it in main text if asked
- #2 Adding a section entitled “implications for management” has also been well received

**C15**

Ecology is the study of the relationships between living organisms, including humans, and their physical environment. But what allowed organisms to establish themselves under these conditions? How does the system change over time?

- To understand how organisms were distributed or how your system changes over time, first, you must understand the variability of the system, to be able to separate the natural variability from the variability due to historical events. For this, it is necessary to use replicas in the study of paleontology.
- A well-supported work that explores the variability of the system, using no simulated (e.g. your data) and simulated data is an attractive option for ecological journals.
- On the other hand, the fact that early-career students have a multidisciplinary group not only made up of geologists or those related to paleontology will allow them to have a broader vision of their work and the possibility of publishing it in different journals.

**C16**

I think that a good approach to reach an audience outside the paleo-scope is

- To explain in a simple way the processes involved in landscapes formation (from tectonics, geology and geomorphology). In my previous experience
- I see people attracted to the potential promotion of interesting landforms, geological formations, soils, and even fossils through geotourism. As you can agree with me, people are looking to profit with knowledge, so tourism is an option to achieve this in a more "sustainable" way.
- Other tips on how to get the editors of journals outside the prefix "paleo' willing to accept your manuscripts with paleo-data for review, it is that the papers should include good images, figures, maps of the reconstruction of the paleoenvironments. For example, when you are in a museum, you love to see good examples of how these environments were, as much vivid as they are, you feel into it. For doing this, you need the help of a visual designer, or a good drawer, if not you can not translate those results into the people's imagination (including editors).

**C17**

I guess I have never had a problem publishing paleo results outside of paleo journal venues. Since my team largely works on the Laurentian Great Lakes we have published several paleo studies in the Journal of Great Lakes Research.

- If I was to give a 'tip' on how that's done, basically it is helpful to have important management implications for the theme of the journal, in this case 'large lakes'. We have used paleolimnology to identify the driving stressors in the lakes, and have made predictions of future conditions. The Great Lakes research community has been very open to our work.
- Some of the other non-paleo journals we've published in are non-specific journals like PLOS ONE and PeerJ. So I suppose using those nonspecific open-access journals is another (if somewhat boring) way to get our research outside of JOPL and the like.
- Something I've done occasionally is intrude upon a new venue when developing an indicator I've never used before. So for instance we are submitting a paper to a cancer journal because we've reconstructed the history of mesothelioma-causing fibers from cores near taconite mines. In other cases we're publishing in taxonomic journals because we do lots of diatom analysis and come up with historical florae and new species, but lots of diatom/paleo folks do that -- Jeff Stone does it lots. And of course lots of paleo researchers are getting into climate journals due to the strong climate signals we see in records, on short and very long scales.

**C18**

- I am not sure if I can be of help here and if I understand your request correctly, but I would say that palaeo studies with a focus on **indicator** value, i.e. studies that show the sensitivity of one or more proxies against a certain or several environmental stressor(s), can be published in journals outside the palaeo-realm.
- Another aspect is that palaeo studies are **long-term** studies and no 'modern' study can give the same information of ecosystem response because of limitation in length/duration of monitoring data. This is in particular important for investigating the effects of climate change (time range: >30 yrs) but also processes that develop over long(er) time scales, e.g. acidification or recovery from eutrophication etc.
- So, high-resolution and multi-proxy palaeo studies have/should have a high value for ecologists in **understanding ecosystems and overall ecological processes** or for socio-ecologists if anthropogenic aspects (disturbance, resilience etc.) are included as well. But I guess you are very aware this, esp. of the latter.

**C19**

I think a few rules that I consider are:

- Consider your results critically. What are they telling you beyond a historical or sediment "story". That bigger picture opens up a myriad of journal opportunities.
- Intensionally link your analyses to hypothesis-driven science--paleo is the tool to address those hypothesis. This will open up your work to broader journal coverage in subjects from climate change, urban ecology, emerging contaminants, global processes, resource management.
- Recognize the broader impact of your work and its interest to broader audiences. Many environmental issues will value from historical perspectives and welcome novel approaches. Further, the use of sediment records and paleolimnology is of interest to much more than science journals and can easily be used to develop outreach, curricula, and experiential activities.

**C20**

As I focus on the last century, I rarely use the term “paleo”. If it helps, I propose some preliminary ideas:

- Design your project based on the potential application of the results to present (and maybe future?) conditions.
- Do not use “paleo” in the title, it sounds “paleo” and too specialized. An option may be “reconstruction”, but many other are possible.
- Target journals related some of the main subjects (e.g. climate, human impact, water quality) or methodologies used (e.g. foraminifera, ecology, geochemistry).
- Target generalist journals.

**C21**

- Palaeoecology should continue its move from being a descriptive discipline to one which specifically tests hypotheses, that can be related to eg ecology, biodiversity, global change etc, thereby widening out the interest scope, but also making for more robust science

- Studies should continue characterizing the uncertainties between target variables and proxy sources to make robust ecological and evolutionary inferences

**C22**

- Have a good research question that is also relevant outside 'palaeo'. It would then be of interest to non-palaeo people who would then hopefully read it.
- Have clear aims and relevance, do excellent research, write it up with non-palaeo in mind. Discuss the wider implications and relevance to non-palaeo and make the conclusions clear and positive.
- Avoid vague statements like - these can inform the future - etc. e.g. for climate, biodiversity, conservation, etc. Be very clear HOW they are relevant and informative. Otherwise, non-palaeo end up by asking What, Why, How? - so what!
- More and more non-palaeo journals are now publishing 'palaeo' papers, e.g. J. Ecol, J. Veg. Sci. Palaeo is already becoming part of main-stream ecology. So this issue is not so much of an issue any more.

**C23**

Proposals:

- Having knowledge for the specific topic for which it wants to communicate to other areas outside the paleontological realm.
- Counting on a suitable paleontological information to treat the topics on focus.
- Utilizing a common and suitable language in order to communicate among areas for better understanding.
- Emphasizing the comparisons and contrasts between paleontological and present data for inferring a better future.
- Highlights and links with dynamic data in the form of graphs, images, QR codes, detailed reconstructions (visual communication).
- That articles which contain ecological data, such as abundance data (eg NTR, NMI, pollen percentages, etc.), are available to people who wish to access them (eg Open access data on Github or other platforms).

**C24**

I’m interested in long-term (century-scale) ecological change, so my work spans between regular ecology and palaeo-research. (I’m also interested in the impact of disturbance events – specifically the deposition of volcanic ash – in the distant past, so I have a foot in the geosciences camp, too).

My research naturally involves inference - I often use chronosequences, for example - and I have encountered some resistance from ecological journals due to the uncertainties involved. Referees accustomed to manipulative experiments/direct observation are often uncomfortable with time-for-space substitution as an approach. Replication is rarely possible, and this is frequently and issue. With palaeo journals, I have had a different problem, in that the temporal and spatial scales of my study appear too fine, compared with work that is typical in this field.

In terms of tips, I would say:

- Understand the interests and methodological approaches of the target audience.
- Stress what your approach can offer that methods more usually used by your audience cannot (and point out the benefits of a holistic approach).
- Be open about methodological limitations of your approach (and do what you can to minimise these).

For example, chronosequence work is not perfect. It makes certain assumptions, but errors can be minimised with careful attention to site selection, dating control and so forth. However, it offers a perspective unavailable by direct observation. And results derived from chronosequences have real relevance for processes that are occurring today, e.g., wildfires. Similarly, contemporary observations of impacts and volcanic ash layers can greatly assist the interpretation of the palaeorecord.

**C25**

I am mostly a passive user of this group here, as I did palaeoecology and peatlands in my master, published a paper on it and then moved on into another field (microclimate and microrefugia)

But I have studied an extremely interdisciplinary programme (landscape ecology + nature conservation), which as such created a good basis for understanding different disciplines. I don't know if it makes it to the top-ten, but simple basic education - high-school and university - can be the start to being able to think broadly and diversely.

As a PhD and now Postdoc in Ecology I have been an active member in the Bolin Centre for Climate Research in Stockholm, which unites researchers from all sorts of disciplines, including historical and deep time climate variations, weather and climate models, glaciology, geology, evolution etc etc.

The common conferences and interdisciplinary projects are always a challenge, because people cannot leave their jargon, and they don't understand each other.

- So, in order to be able to talk, write and publish for a broader audience, you need to learn to think like them. You learn this, by being among them, chatting with them. Going on excursions with them. As a student this is rather easy. A summer school from this climate centre forced PhD students to work on different topics in mixed groups and one got good insight into other disciplines.

I also participated once in a workshop on science collaboration and networking, where the final big task was to develop a common research proposal in a group of 5-6 students from different disciplines. The proposal should be a novel and relevant idea that also built on our diverse expertise. It was challenging and great fun and you were forced to understand what the core qualities of the other researchers and disciplines were. Which parts they could contribute. And this can lead to collaboration, which can lead to publication in other journals.

- Also, writing in a concise, entertaining and exciting way is another wild card to get good reviews from journals outside your field. Having a good story and having the language to convey this story.

For example, I loved Guthrie, 2001, which waves together insights from different disciplines to explain a simple palaeoecological theory.

(<https://www.sciencedirect.com/science/article/pii/S0277379100000998?casa_token=7sDxHKbO65IAAAAA:mP7jLoYiQxP97sDgrqbZuRqjPo6Y-qrJmqj_0UZuo8kB0rBc7i-KQRTUQntCHvRdNPQX60EmPsM>).

- I guess, if one wants to publish in other journals, a good idea is start reading something from them and reading abstracts of other disciplines, so one gets a feeling of where his/her own research fits in and to which other ongoing research threads it connects.

**C26**

- One tip I had is "connect paleo-climate to modern climate change and its ramifications."

**C27**

Since most (not all) of my work is on paleolimnology over the last ~200 or so years, this has not really been an issue. It has been fairly easy to get papers into non-paleo journals, as we are addressing issues that are “on the radar screen”. For example, recent algal blooms, all aspects of climate change, mining impacts, eutrophication, exotic species, and so forth.

So the key questions I use there are:

- Have conditions changed? If so, when and why? What were pre-impact (i.e. reference) conditions? (realistic targets for mitigation).
- Without direct monitoring data the only avenues we have left are historical documents of some kind (like diaries), indigenous knowledge, and paleo approaches.

At least working on time scales of recent human impacts (like last 200 or so years), this is fairly easy to get across to non-paleo journals and readers.

But “recent paleolimnology” may be an exception with overall paleo studies on more esoteric topics and on longer time scales being more of a challenge. But the longer the time frame, the better understanding we have of natural modes of environmental change, and can compare those to the “Anthropocene” period.

**C28**

- Rule 1: Entering application-related research in “Paleo” geoscience

It is important to enter specific areas where “paleo” science research can be applied directly such as petroleum geoscience and mineral exploration. For example, the study of geochemistry of sedimentary rocks can be used to evaluate source rock potential, hydrocarbon generation potential, and maturity as well as paleoclimate of oceanic and continental sedimentary basins. In addition, paleo flora/palynofacies analysis can also be used in petroleum research. Such studies can be easily published in journals like Marine and Petroleum Geology, Journal of Petroleum Exploration and Production Technology, Organic Geochemistry, American Association of Petroleum Geologists Bulletin, Petroleum Science, etc. Specifically, the scope of Marine and Petroleum Geology (impact factor = 4.348) is “essential reading for geologists, geophysicists and explorationists in industry, government and academia working in the following areas: marine geology; basin analysis and evaluation; organic geochemistry; reserve/resource estimation; seismic stratigraphy; thermal models of basic evolution; sedimentary geology; continental margins; geophysical interpretation; structural geology/tectonics; formation evaluation techniques; well logging”. However, in this journal, the find articles with the term “paleo” are linked with 198 publications in 2021, 210 publications in 2020, and 140 publications in 2019.

The analysis of sediment cores can also be used to study paleoclimate and mineral enrichment. For example, such studies can be focused on mineral exploration, and can be published in the Journal of Geochemical Exploration, International Journal of Sediment Research, etc. Specifically, the Journal of Geochemical Exploration (impact factor = 3.746) is “mostly dedicated to publication of original studies in exploration and environmental geochemistry and related topics”. However, in this journal, the find articles with the term “paleo” are linked with 18 publications in 2021, 15 publications in 2020, and 21 publicatiRule 1: Entering application-related research in “Paleo” geoscience

It is important to enter specific areas where “paleo” science research can be applied directly such as petroleum geoscience and mineral exploration. For example, the study of geochemistry of sedimentary rocks can be used to evaluate source rock potential, hydrocarbon generation potential, and maturity as well as paleoclimate of oceanic and continental sedimentary basins. In addition, paleo flora/palynofacies analysis can also be used in petroleum research. Such studies can be easily published in journals like Marine and Petroleum Geology, Journal of Petroleum Exploration and Production Technology, Organic Geochemistry, American Association of Petroleum Geologists Bulletin, Petroleum Science, etc. Specifically, the scope of Marine and Petroleum Geology (impact factor = 4.348) is “essential reading for geologists, geophysicists and explorationists in industry, government and academia working in the following areas: marine geology; basin analysis and evaluation; organic geochemistry; reserve/resource estimation; seismic stratigraphy; thermal models of basic evolution; sedimentary geology; continental margins; geophysical interpretation; structural geology/tectonics; formation evaluation techniques; well logging”. However, in this journal, the find articles with the term “paleo” are linked with 198 publications in 2021, 210 publications in 2020, and 140 publications in 2019.

The analysis of sediment cores can also be used to study paleoclimate and mineral enrichment. For example, such studies can be focused on mineral exploration, and can be published in the Journal of Geochemical Exploration, International Journal of Sediment Research, etc. Specifically, the Journal of Geochemical Exploration (impact factor = 3.746) is “mostly dedicated to publication of original studies in exploration and environmental geochemistry and related topics”. However, in this journal, the find articles with the term “paleo” are linked with 18 publications in 2021, 15 publications in 2020, and 21 publications in 2019.

- Rule 2: Focusing “Paleo” science research to specific regional/geographical areas

Paleoclimate research can be focused on specific geographical regions, and such studies can be published in the journal like Journal of Asian Earth Sciences, Journal of African Earth Sciences, Arabian Journal of Geosciences, Regional Studies in Marine Science, etc. Specifically,

the Journal of Asian Earth Sciences (impact factor = 3.449) is “an international interdisciplinary journal devoted to all aspects of research related to the solid Earth Sciences of Asia. The Journal publishes high quality, peer-reviewed scientific papers on the regional geology, tectonics, geochemistry and geophysics of Asia”. However, in this journal, the find articles with the term “paleo” is linked with 74 publications in 2021, 147 publications in 2020, and 147 publications in 2019.

- Rule 3: Focusing “Paleo” science research on specific environmental phenomena

Paleo research can be focused on specific environmental phenomena, and such studies can be published in journals having entirely different scopes. For example, several research articles related to paleo-tsunami deposits were published in a journal like Natural Hazards (impact factor = 3.102) which has no scope related to paleo. Natural Hazards has the scope of “all aspects of natural hazards, including the forecasting of catastrophic events, risk management, and the nature of precursors of natural and technological hazards”.

**C29**

- Do not think of paleoecology as a discipline, but as a toolbox to address a wide range of ecological research questions requiring long-term perspective.
- Make a habit of reading publications (not only the Abstracts...) in the non-paleo journals in which you want to publish, to get a feel for which type of research questions are within the scope of those journals, and how they are typically addressed.
- Make sure the length, resolution and dating potential of your paleorecord is in accordance with the research question being addressed, so that the proxy data can be reliably considered as long time series of ecological monitoring data. If your site fails these criteria, either look for a better site; or address a different question, one that can be robustly addressed by the available paleorecord.
- Develop the paleostudy, and resulting publication, with emphasis on the (non-site-specific) larger research question that is being addressed, not on the paleoecology tool-box that is being used for this purpose.

**C30**

From my perspective, one of the most important points is to make research interesting to people outside our field. How can we do this?

- For instance, first of all we have a problem with the way we plot the data, our beloved stratigraphic diagrams, they are boring and poorly informative to other professionals.
- Equally important is to make clear the relevance outside palaeo. For example, mostly all palaeo papers argue that "this data will be valuable for conservation or fighting against climate change", but rarely none says how or even takes the step forward and shows it applied.
- Sometimes the problem is that we (the palaeo-community) continue doing the same (explicit palaeo-papers) and demand or desire other people being interested, because "what we do is so cool and everyone should recognise its value". We need to tackle the other way around: when I am working with a palm species, I write about palm population or community dynamics, incorporating a long-term view.

I am quite sure you already know all this, but being generic without specific examples I would start from here. I am happy to chat at any time.

**C31**

Your topic is a vital one, given the pressures placed upon nature, and one I find myself wondering with each manuscript.

- I think something along the lines of ‘providing directly-transferable numerical estimates of rates and thresholds (where appropriate), with confidence bounds’.

**C32**

I have to admit that I am somewhat of a newcomer to the paleoecology field. I actually started working the other way around, convincing paleooceanographers that they should care about ecology, but I am now also trying to go the other way (paleo to modern).

That said, I have actually not had that many problems with getting my research published outside of the paleo domain. Perhaps this is because as paleoceanographer/paleoclimatologist I am a bit more used to linking with the modern oceanography/climatology community? In any case, the points I try to emphasise to make my research more relevant is

- that the paleo record can provide insights on time scales that we simply can’t observe with “normal ecological time series” and
- that the paleo record can provide a baseline of natural ecosystem variability prior to human influence.
- Another relevant point is that I (or my co-authors and I) tried to explicitly link paleo observations with modern observations. I hope this is useful, but maybe these points are trivial, so feel free to ignore them. In any case, I’d be happy to hear how this project evolves.

**C33**

This is definitely both a real issue and something important to discuss. There are a lot of issues, and I’m not quite sure how to distill my suggestions down to tips…

- Having a very clear hypothesis/aim which is focused on non-palaeo questions helps, as does focusing methods text on the justification of why the method chosen is going to produce relevant data (so essentially starting methods explanations a step back in the chain of reasoning than we’d use in a palaeo paper).
- Showing very clear understanding of the spatial and temporal resolution and taxonomic and taphonomic limits of the data, which is a good basis for arguing for what it CAN show, is useful – palaeoecologists are not very good in my experience at addressing these things in papers to their own community, either because they don’t really think about them and follow internalised conventions or because they assume everyone is aware of them so it would be boring, and it’s something we could to do better in house as well as externally. I guess those things come down to pre-empting some of the criticisms that neoecology often raises against palaeoecology…

- Also putting more time into the abstract than you might normally, to make sure it is really, really problem focused and problem answering.

- And for journals that still accept them writing a cover letter which makes a positive case for your chosen approach and it’s relevance to the journal, using words and phrases from its stated aims and purpose and audience – are important in my experience for getting the paper sent out to referees rather than desk-rejected, and then for persuading referees who aren’t certain to ask for revisions not rejection, as well as once it’s published for communicating with readers (for all published articles, far more readers skim the abstract and conclusions than actually read the whole thing line by line, and that’s fine and normal, but the editors and referees DO go line by line so getting those things right first time round matters, especially when going outside your “natural lane” of publication).

**C34**

I wasn't a paleo person originally; my PhD is in an engineering school and the focus is on water resources. I just discovered tree rings along the way. And when I do these reconstruction works, my focus was always to make them relevant to water resources, and the papers were written with that in mind.

As I am now working with a historian, I experience how hard it is to talk to an audience outside the discipline. I didn't experience this when writing for WRR because I am familiar with the way WRR papers are written.

So my tips would be as follows (which are rather common sense)

- Know your audience. If you want to publish in an outside journal, make sure you know who the audience is and what they expect, and write accordingly. This could be very different from how you would normally write. My papers are very different from one you would see from Dendrochronologia, for example.
- Collaborate with someone from that field. They will help you achieve tip #1 above. When we wrote our proposal my historian colleague helped a lot in tweaking my language so that I don't sound like an alien to historians. They will help you choose an appropriate journal. Case in point: having a water resources background, I know that I have much better chances publishing in WRR than in Hydrological Sciences Journal based on my past readings of these journals. And also, having an insider co-author will help generate a sense of familiarity in readers and attract them to your paper more.

**C35**

- I believe a crucial point is to use terminology as close as possible to the target discipline and clearly explain vocabulary that is not commonly used even if it is a standard term in your own field: for example, a historian will struggle with time scales in "yr cal BP", an ecologist will need some help in understanding words like "palynology"
- Try to adapt methods to a frame that is similar what the target discipline does: for example, my ice core colleagues appreciate me running some replicate samples along the record, giving uncertainty bars for charcoal and pollen concentrations etc as these are standard procedures in many disciplines but often not applied in traditional pollen studies due to our established methods.
- Conduct research on open questions in the research field you want to publish in rather than try to fit your paleoecological record afterwards into a e.g. modern ecology framework so your research design fits the focus from the beginning
- Simplify figures to essential information, none other than paleoecologists understand a figure with more than 5 curves for species. The rest of data can be presented in supplementary material
- Consider adding a conceptual figure explaining the approach, the processes to be studied or summarizing the main results in an appealing way.
- Get an expert in the field as a co-authors, they give valuable feedback, help to adjust the language of the manuscript and also make a study more credible in their own field as it is not just "an outsider" trying to publish in the field
- Check out the style of other paleocology studies that published previously in the target journal
- Make sure you capture the relevant research of the discipline (your expert co-author may help you here as well if you overlook an important paper of that field)

**C36**

- Read read read.

Getting familiar with the journal’s jargon, terminology closest to your wording, and research questions of interest is of essential value to understand how to rephrase your “paleoecological” wording to wording that matches the background of the journal you are aiming for. Getting a good set of exemplary papers from the journal of interest, relatively close to your topic, can help give ideas on the terminology you likely want to adapt to your paper as well. Also, get a good overview of research questions that have been addressed over the course of the last 2 years in the journal of interest and how these have been framed. On which topics did they compile Special Issues? Were research questions addressed more conceptually or more focused on statistical analyses? Read a good set of papers from your journals of interest; nothing wrong with identifying exemplary papers and use them as a template if you are not sure what structure to follow.

- Keep the message simple

In paleoecological research, studies tend to try to describe everything they can from the record obtained. To publish in other journals, the style best to approach is to keep the message of your paper simple. Avoid describing all proxies and time periods not specifically needed for the main key message you try to convey. Keep terminology consistent and simple throughout the paper, and make sure that all key concepts are clearly defined. When an editor or reviewer pass too many misunderstood words, they will lose the main message of the paper, and they can easily get de-motivated for a supportive review.

- Focus on the research question, not the proxy

Identify one key research question to build your paper around (surely “smaller” ones can be addressed in the paper, but better to have one clear cut stand out), and make sure that you describe in simple but exciting wording how your proxy is useful to address this research question. The research question should match the interest of the journal and doing your home-work in reading in therefore essential.

- Consider a conceptual figure

Never a dull moment with a good conceptual figure! The level of attractiveness of your paper can increase substantially by adding a conceptual figure, if appropriate; even a small one will likely be much appreciated by the editor and reviewers. When targeting a journal outside your field, it can increase your chances that your paper is well understood and therefore well received. Present the key terminology of your paper in the conceptual figure and stick to that terminology throughout your paper.

- Go smart on the technical details

You will likely want to carefully craft the technicalities of your methods. Be sure that you do not engage in too many technical details on the description of the proxy (e.g. pollen, diatoms, charcoal, etc), so that your paper won’t be read as “here’s a paleoecological paper trying to do some other research”. The method section in a paleoecological journal is likely to be different to such section in a journal in another field, so put effort in making an attractive description understandable for a wide audience.

- Share with close colleagues outside your field

If you have time, share your manuscript with supportive colleagues slightly outside your field, so that they can help you identify the sections that are unclear, too technical, or where they see most work needed. This can be specifically useful also in an earlier stages of the idea development of your manuscript, perhaps by presenting your ideas to an audience with different backgrounds and see what they pick up on as the most interesting ideas and results.

- Get yourself in by a good cover letter to editor

Use the cover letter to show that you did a thoroughly analysis of why you think that your paper fulfils the interests of the journal. Share the cover letter with colleagues outside your field and see if your message is convincing. Even better, if you might know one of the editors of the journal, reach out and ask for advice up front. You can also briefly describe in the cover letter what opportunities might open up in the future once your particular study is published, and why this would be of particular interest for the journal. This kind of information might not be appropriate for the paper itself, but can give an editor from a different background more context about where the field might go in the future. Getting the editor excited for your work is as critical step as getting your manuscript in the right shape, so don’t underestimate the time needed to get it ready and convincing.

- Consider a cross-disciplinary co-author team

Consider having a non-specialist colleague on board of your co-author list who can keep track of wording and technical sections as they develop, and exciting and unexpected ideas might pop up! Also colleagues experience in publishing in the journal of interest might give you key insights in expectations of the journal in terms of content and style, and likewise can help keep the wording in line.

**C37**

- One of the things I found very related to eco-paleo approaches are modern analogues and how we can hindecast conditions through them. We can test hypotheses such as intermediate disturbance (Grime 1973) using paleo records. I briefly touched upon this with sedDNA over the Holocene and human impact gradients with plants.

**C38**

- Do your homework: make sure you know what the priorities and interests of your target audience are and how they approach them. This should go beyond just reading the aims and scope of the journal, and include reading and, ideally hearing from others in your non-palaeo discipline or field before you write your paper, e.g. attend online talks by relevant organisations/individuals, join seminars or discussion groups in other departments in your institution to listen and ask questions.
- If your non-palaeo target is an applied journal (e.g. conservation, environmental or heritage management), familiarise yourself with the practical challenges they face so you don’t make it an effort for them to see the relevance of your evidence. If examples are allowed, I like the way that Morales-Molino et al. 2019 suggest practical ways that their data on long-term herbivory can be used in management in the final lines of the conclusion: <https://esajournals.onlinelibrary.wiley.com/doi/abs/10.1002/ecy.2833>
- Be honest about the limitations of your discipline from their perspective: this can be challenging to write, but avoids an editor/reviewer using this to undermine your effort. Be aware of how non-palaeo papers express the limitations of palaeo. All disciplines have limitations so identifying ways that palaeo can address potential weaknesses in other disciplines and also how ecology informs palaeo can allow your work to connect with sources that are familiar and provide a strong argument about how complementary insights can emerge by combining different lines of evidence.
- Stand on the shoulders of giants: many others have thought about and achieved this, so find good role models. How do they express the relevance of palaeo to other fields? How have papers on this topic been cited by non-palaeo writers? Palaeo-scientists who are editors on non-palaeo journals may able to offer ‘inside’ insight.

**C39**

- Applied research has the best prospect of being published in non-paleo journals, so focus on the applied aspects.
- Having a co-author who is a practitioner or end-user in the field (e.g. a field ecologist) helps, so get at least one on board.
- For a first foray, choose a non-paleo journal that has previously published paleo research (yes, they do exist!).
- A 'Methods' paper (e.g., one that uses paleo methods in a novel way for contemporary ecologists/geoscientists) could be a good angle, but the application does have to be novel and reproducible.
- Make clear in the manuscript the added insight to contemporary issues/problems from [application of] paleo data/methods.
- Most (though not all) paleo datasets will need re-formatting/re-presenting for a non-paleo audience; use graphical /tabular presentation familiar to readers of the target journal.
- The Title and Abstract are crucial: they must make clear the [contemporary/practical] applications of the paleo research.
- The cover letter to the Journal Editor must make very clear the relevance to the journal's readership, and why it should be published in the journal.
- Ensure that citations are made within the manuscript to relevant papers published in the target journal.
- If Reviewers are requested by the journal, ensure that both the following are included: (a) non-paleo scientists who have made use of paleo data and so who are likely to be sympathetic to the approach; (b) paleo scientists who are well known for publishing in non-paleo journals.
